# Radiosynthesis and *in Vivo* Evaluation of Two PET Radioligands for Imaging α-Synuclein

**DOI:** 10.3390/app4010066

**Published:** 2014-03-17

**Authors:** Xiang Zhang, Hongjun Jin, Prashanth K. Padakanti, Junfeng Li, Hao Yang, Jinda Fan, Robert H. Mach, Paul Kotzbauer, Zhude Tu

**Affiliations:** 1 Department of Radiology, Washington University School of Medicine, 510 S. Kingshighway Boulevard, St. Louis, MO 63110, USA; 2 Department of Neurology, Washington University School of Medicine, 660 S. Euclid Ave, St. Louis, MO 63110, USA

**Keywords:** Lewy bodies, Parkinson's disease, PET, phenothiazine, radiosynthesis, α-synuclein

## Abstract

Two α-synuclein ligands, 3-methoxy-7-nitro-10*H*-phenothiazine (**2a**, *K_i_* = 32.1 ± 1.3 nM) and 3-(2-fluoroethoxy)-7-nitro-10*H*-phenothiazine (**2b**, *K_i_* = 49.0 ± 4.9 nM), were radiolabeled as potential PET imaging agents by respectively introducing ^11^C and ^18^F. The syntheses of [^11^C]**2a** and [^18^F]**2b** were accomplished in a good yield with high specific activity. *Ex vivo* biodistribution studies in rats revealed that both [^11^C]**2a** and [^18^F]**2b** crossed the blood-brain barrier (BBB) and demonstrated good brain uptake 5 min post-injection. MicroPET imaging of [^11^C]**2a** in a non-human primate (NHP) confirmed that the tracer was able to cross the BBB with rapid washout kinetics from brain regions of a healthy macaque. The initial studies suggested that further structural optimization of [^11^C]**2a** and [^18^F]**2b** is necessary in order to identify a highly specific positron emission tomography (PET) radioligand for *in vivo* imaging of α-synuclein aggregation in the central nervous system (CNS).

## 1. Introduction

Although Parkinson's disease (PD) is a degenerative neurological disorder characterized by motor symptoms, it is also known to be closely associated with dementia [1]. The primary neuropathologic change in PD, the degeneration of dopaminergic neurons, occurs in the substantia nigra, accompanied by Lewy bodies (LB) and Lewy neurites (LN). To date, the pathogenic mechanism of PD is not fully understood [2]. The diagnosis of PD is primarily based on the clinical symptoms, such as resting tremor, bradykinesia and rigidity. Because the current treatment for PD is to minimize the disease symptoms in the patients [1, 3], a method of diagnosing PD at a very early stage would greatly help physicians to design the therapy accordingly.

α-Synuclein (α-syn) is a presynaptic terminal protein that consists of 140 amino acids; the aggregation of α-syn is considered the pathological hallmark of PD. α-Syn plays an important role in the central nervous system (CNS) in synaptic vesicle recycling; it also regulates the synthesis, storage and release of neurotransmitters [4]. It is specifically upregulated in a discrete population of presynaptic terminals of the brain during acquisition-related synaptic rearrangement [5]. α-Syn naturally exists in a highly soluble, unfolded state [6,7]. However, in PD brains, insoluble aggregation of misfolded fibrillar α-syn occurs in LB and LN, which may cause synaptic dysfunction and neuronal cell death [8–11]. Positron emission tomography (PET) is a non-invasive imaging modality that can provide the functional information of a living subject at the molecular and cellular level. Current diagnostic PET radioligands for PD target either the dopaminergic system (pre-synaptic and post-synaptic dopamine activity) or vesicular monoamine transporter type 2 (VMAT2) [12,13]. Unfortunately, such imaging strategies have difficulty in distinguishing PD from other parkinsonian syndromes that also result in the degeneration of nigrostriatal projections [14,15]. In addition, dopaminergic medications used for symptomatic treatment may alter striatal uptake of these agents, limiting their reliability for measuring disease progression [16]. In contrast, α-syn is a valuable imaging target for PD, because fibrillar α-syn deposition in LB and LN distinguishes PD from other disorders and is the defining feature for post-mortem pathologic diagnosis. Thus, a small molecular PET radiotracer with high affinity and selectivity to fibrillar α-syn protein could be used to quantify the level of α-syn aggregation non-invasively. This will not only improve the diagnostic accuracy of PD, but also provide a tool to improve the understanding of disease progression and monitor the therapeutic efficacy in clinical trials.

**Figure 1:**
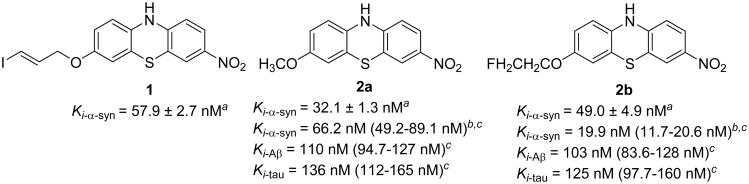
Potent tricyclic aromatic ring analogues. a Thioflavin T fluorescence assay; b ^125^I competitive binding assay; c 95% confidence intervals for *K*_i_ values are shown in parentheses.

**Figure 2:**
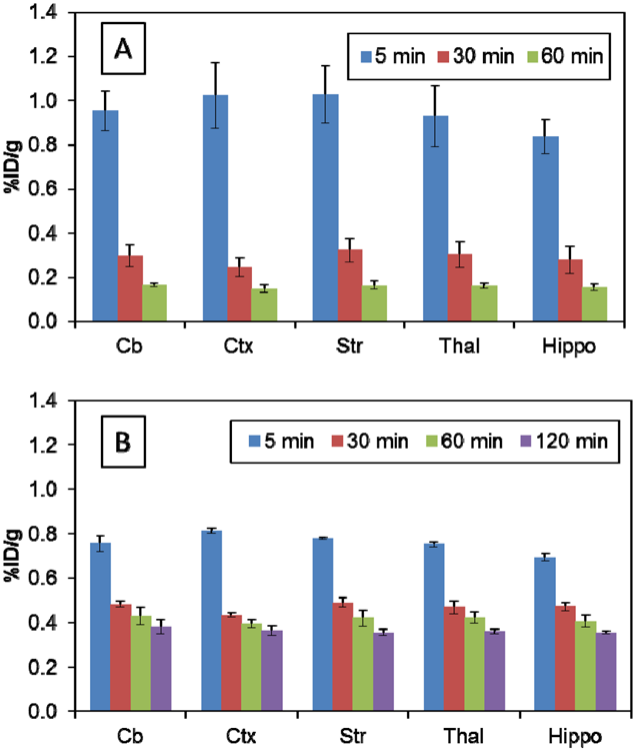
Regional radioactivity uptake in male Sprague Dawley (SD) rat brain (*n* = 4). (**A**) Regional brain uptake of [^11^C]**2a**; (**B**) regional brain uptake of [^18^F]**2b**. Cb, cerebellum; Ctx, cortex; Str, striatum; Thal, thalamus; Hippo, hippocampus.

Our group previously reported the syntheses of a class of tricyclic analogues and their *in vitro* binding affinities towards α-syn fibrils; several lead compounds were identified with moderate affinities for α-syn fibrils (*K_i_* < 70 nM) (Figure 1, **2a**, **2b**) [17]. Compounds **2a** and **2b** also displayed favorable binding selectivity to α-synuclein aggregation compared to Aβ and tau protein: for **2a**, *K_i_*_-α-syn_/*K_i_*_-Aβ_ > 3-fold and *K_i_*_-α-syn_/*K_i_*_-tau_ > 4-fold; for **2b**, *K_i_*_-α-syn_/*K_i_*_-Aβ_ = 2.1-fold and *K_i_*_-α-syn_/*K_i_*_-tau_ = 2.5-fold [18]. The radioiodinated ligand, [^125^I]**1**, was synthesized to establish a methodology for screening the α-syn fibril binding affinity of new ligands using a competition binding assay [18]. The affinities for **2a** and **2b** were determined using this [^125^I]**1** assay, and the resulting *K_i_* values (66.2 nM for **2a**, 19.9 nM for **2b**) were in the same range as the values obtained by the Thioflavin T assay. The ^125^I competition assay further confirmed the previously determined *in vitro* potency of **2a** and **2b**, which were developed as potential PET radioligands to be radiolabeled by ^11^C or ^18^F. In the current manuscript, we report the radiosyntheses of [^11^C]**2a** and [^18^F]**2b** and their validation in animal studies to determine whether [^11^C]**2a** and [^18^F]**2b** can penetrate the blood-brain barrier (BBB), have sufficient brain uptake and fast washout from the brain. Results of *ex vivo* biodistribution of [^11^C]**2a** and [^18^F]**2b** in Sprague-Dawley rats and microPET CNS imaging in a cynomolgus macaque of [^11^C]**2a** suggest that further structure-activity relationship (SAR) study is necessary for identifying a highly specific PET radioligand targeting α-syn aggregation.

## 2. Experimental Section

### 2.1. Chemistry

#### 2.1.1. General

All reagents and chemicals were purchased from Sigma-Aldrich Corporation (Milwaukee, WI, USA) and used without further purification, unless otherwise stated. The melting points of all intermediates and final compounds were determined on a Haake-Buchler melting point apparatus and are uncorrected. ^1^H and ^13^C NMR spectra were recorded on a Varian-400 MHz spectrometer (Varian Inc. Palo Alto, CA, USA), which is maintained by the Chemistry Department of Washington University in St. Louis, MO, USA. Spectra are referenced to the deuterium lock frequency of the spectrometer. The chemical shifts (in parts per million) of residual solvents were found to be at 7.26 for CHCl_3_ and at 2.50 for dimethyl sulfoxide (DMSO).

**Scheme 1:**
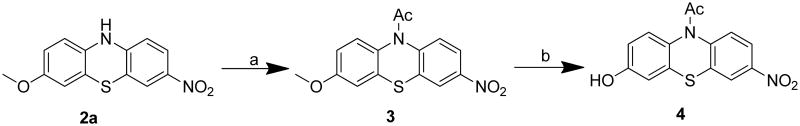
Synthesis of phenol Precursor **4**. rt, room temperature. Reagents and conditions: a AcCl, DCM, rt, overnight; b BBr_3_ in DCM (1.0 M), DCM, −78 °C–rt, overnight.

Compound **4**, the precursor for radiolabeling [^11^C]**2a** and [^18^F]**2b**, was synthesized according to Scheme 1 and the previously reported procedure [17] with necessary modification.

#### 2.1.2. 1-(3-Methoxy-7-nitro-10*H*-phenothiazin-10-yl)ethanone (**3**)

Acetyl chloride (360 mg, 4.59 mmol) was added into the solution of Compound **2a** (420 mg, 1.53 mmol) in dichloromethane (10 mL). The reaction mixture was stirred overnight at room temperature (rt). The solvent and excess acetyl chloride was removed under reduced pressure. The residue was dissolved in ethyl acetate and washed with water and saturated sodium chloride solution. The organic extract was dried over anhydrous Na_2_SO_4_ and purified by silica gel column chromatography using ethyl acetate/hexane (1/2, *v*/*v*) as the mobile phase to yield Compound **3** as a yellow solid (267 mg, 55%). ^1^H NMR (CDCl_3_): δ 2.23 (s, 3H), 3.83 (s, 3H), 6.90 *(d, J* = 9.0 Hz, 1H), 6.98 (s, 1H), 7.32 (*d, J* = 8.7 Hz, 1H), 7.72 (d, *J* = 8.7 Hz, 1H), 8.18 (d, *J* = 8.7 Hz, 1H), 8.29 (s, 1H). ^13^C NMR (CDCl3): δ 22.9, 55.7, 112.7, 114.0, 122.0, 122.9, 127.4, 127.9, 130.7, 133.2, 134.3, 144.7, 145.6, 158.5, 169.2. Combustion elemental analysis (anal.) calculated (calcd.) for C_15_H_12_N_2_O_4_S: C, 56.95; H, 3.82; N, 8.86. Found: C, 56.72; H, 3.89; N, 8.70. mp 155.9-156.8 °C.

#### 2.1.3. 1-(3-Hydroxy-7-nitro-10*H*-phenothiazin-10-yl)ethanone (**4**)

A solution of BBr_3_ in dichloromethane (1.0 M, 4.2 mL) was added dropwise to a solution of Compound **3** (267.3 mg, 0.84 mmol) in dichloromethane (15 mL) at −78 °C. The reaction solution was stirred overnight at rt. The solvent was removed under reduced pressure. The residue was partitioned between ethyl acetate and water. The organic extract was dried over anhydrous Na_2_SO_4_ and purified by silica gel column chromatography using ethyl acetate/CH_2_Cl_2_ (1/10, *v*/*v*) as the mobile phase to yield Compound **4** as a yellow solid (207.4 mg, 81%). ^1^H NMR (DMSO-d6): δ 2.15 (s, 3H), 6.82 (d, *J* = 9.0 Hz, 1H), 6.93 (s, 1H), 7.47 (d, *J* = 9.0 Hz, 1H), 7.82 (d, *J* = 9.0 Hz, 1H), 8.22 (d, *J* = 9.0 Hz, 1H), 8.39 (s, 1H), 10.00 (br s, 1H). ^13^C NMR (DMSO-d6): δ 23.0, 114.3, 115.3, 122.6, 123.2, 128.4, 128.5, 129.3, 132.3, 134.2, 145.1, 145.6, 156.7, 169.1. High resolution mass spectrometry (HRMS, ESI): *m/z* calcd. for C_14_H_10_N_2_O_4_S [M + 1] 303.0440. Found: 303.0435. Purity: 98% (HPLC confirmed). mp 202.3–205.1 °C.

### 2.2. Radiochemistry

#### 2.2.1. Radiosynthesis of [^11^C]**2a**

##### 2.2.1.1. Production of [^11^C]CH_3_I

Briefly, [^11^C]CH_3_I was produced from [^11^C]CO_2_ using a GE PETtrace MeI Microlab (GE Healthcare, Fairfield, CT, USA). Up to 51.8 GBq of [^11^C]CO_2_ is produced from Washington University's Japan Steel Works BC-16/8 cyclotron by irradiating a gas target of 0.2% O_2_ in N_2_ for 15–30 min with a 40 μA beam of 16 MeV protons. The GE PETtrace MeI Microlab coverts the [^11^C]CO_2_ to [^11^C]CH_4_ using a nickel catalyst (Shimalite-Ni, Shimadzu, Japan P.N.221-27719) in the presence of hydrogen gas at 360 °C; it is further converted to [^11^C]CH_3_I by reacting with iodine held in a column in the gas phase at 690 °C. Approximately 12 min after the end of bombardment (EOB), several hundred millicuries of [^11^C]CH_3_I was delivered as a gas to the hot cell, where the radiosynthesis was accomplished.

##### 2.2.1.2. Radiosynthesis of [^11^C]**2a**

**Scheme 2:**
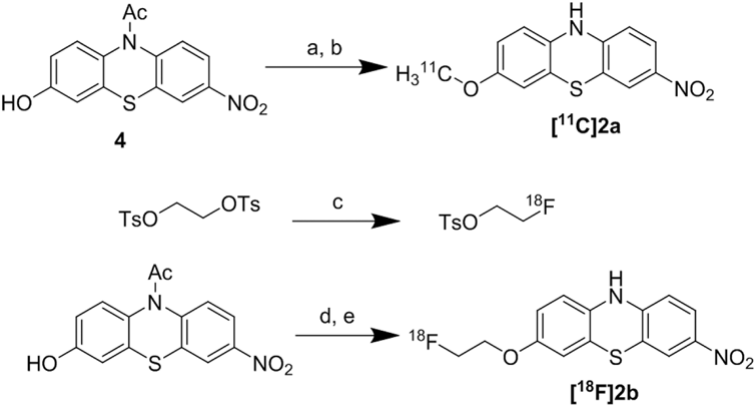
Synthesis of [^11^C]**2a** and [^18^F]**2b**. DMF, *N*,*N*-dimethylformamide; [^18^F]FEOTs, [^18^F]fluoroethyltosylate; DMSO, dimethyl sulfoxide; DBU, 1,8-diazabicyclo[5.4.0]undec-7-ene. Reagents and conditions: a [^11^C]CH_3_I/DMF, NaOH, 90 °C, 5 min; b DBU, 7 min; c [^18^F]F^−^, Kryptofix 2.2.2, K_2_CO_3_, CH_3_CN, 110 °C, 10 min; d [^18^F]FEOTs, Cs_2_CO_3_, DMSO, 90 °C, 15 min; e DBU, 15 min.

Approximately 1.2 mg of Precursor **4** was placed in the reaction vessel, and 0.20 mL of DMF was added, followed by 3.0 μL of 5 N NaOH. The mixture was thoroughly mixed on a vortex for 30 s. A stream of [^11^C]CH_3_I in helium was bubbled for 3 min into the reaction vessel. The sealed vessel was heated at 90 °C for 5 min, at which point the vessel was removed from heat and 20 μL 1,8-diazabicyclo[5.4.0]undec-7-ene (DBU) in 50 μL DMF was added via syringe. The reaction mixture was heated at 90 °C for 7 min (Scheme 2); then, the reaction was quenched by adding 1.7 mL of the HPLC mobile phase, which was composed of acetonitrile/0.1 M ammonium formate buffer (60/40, *v*/*v*) and pH = 4.5. The diluted solution was purified by high performance liquid chromatography (HPLC) by injection on a Phenomenex Luna C18 reverse phase column (9.4 × 250 mm) using the mobile phase described above. The radiolabeled product was eluted using a flow rate of 4.0 mL/min, and the UV wavelength was set as 254 nm. Under these conditions, the retention time of Precursor **4** was ∼7 min; the retention time of [^11^C]2a was ∼16 min. [^11^C]**2a** was collected in a vial containing 50 mL Milli-Q water, which was then passed through a Sep-Pak Plus C18 cartridge (Waters, Milford, MA, USA). The trapped product was eluted with ethanol (0.6 mL) followed by 5.4 mL 0.9% saline. After sterile filtration, the final product was ready for quality control (QC) analysis and animal studies. QC was performed on a Phenomenex Prodigy C18 reverse phase analytic HPLC column (250 mm × 4.6 mm, 5 μA) and UV detection at a 254 nm wavelength. The mobile phase was acetonitrile/0.1 M ammonium formate buffer (80/20, *v*/*v*) using a 1.5 mL/min flow rate. Under these conditions, the retention time of [^11^C]**2a** was 4.82 min. The radioactive dose was authenticated by co-injection with the cold standard Compound **2a**. Radiochemical purity was >99%; the chemical purity was >95%; the labeling yield was 35%–45% (*n* = 4, decay corrected to EOB), and the specific activity was >363 GBq/μmol (decay corrected to EOB, *n* = 4).

#### 2.2.2. Radiosynthesis of [^18^F]**2b**

##### 2.2.2.1. Production of [^18^F]fluoride

[^18^F]Fluoride is produced by a ^18^O(*p*, n)^18^F reaction through proton irradiation of enriched ^18^O water (95%) using Washington University's RDS-111 cyclotron (Siemens/CTI Molecular Imaging, Knoxville, TN, USA). [^18^F]Fluoride is first passed through an ion-exchange resin and then eluted with 0.02 M potassium carbonate (K_2_CO_3_) solution.

##### 2.2.2.2. 2-[^18^F]Fluoroethyltosylate

A sample of approximately 5.55 GBq [^18^F]/fluoride was added to a reaction vessel containing Kryptofix 222 (6.5–7.0 mg). The syringe was washed with 2 × 0.4 mL of ethanol. The resulting solution was evaporated under nitrogen flow with a bath temperature of 110 °C. Acetonitrile (3 × 1.0 mL) was added to the mixture, and water was azeotropically removed by evaporation. After all the water was removed, 5.0–5.5 mg of 1,2-ethylene ditosylate was dissolved in acetonitrile (200 μL), and the solution was transferred into the reaction vessel containing [^18^F]fluoride/Kryptofix 222/K_2_CO_3_. The reaction vessel was capped and the reaction mixture briefly mixed and then subjected to heating in an oil bath that was preheated to 110 °C for 10 min (Scheme 2).

After heating for 10 min, the reaction mixture was diluted with 3.0 mL of the HPLC mobile phase (50/50 acetonitrile/0.1 M ammonium formate buffer, pH = 6.5) and passed through an alumina neutral Sep-Pak Plus cartridge (Waters Corporation, Milford, MA, USA). The crude product was then loaded onto an Agilent SB-C18 semi-preparative HPLC column (250 mm × 10 mm) with a UV detector set at 254 nm. The HPLC system used a 5 mL injection loop. At a 4.0 mL/min flow rate, the retention time of the product was 9.5–10 min. The retention time of the precursor was 23–24 min. The radioactivity peak observed on HPLC was collected and diluted with 50 mL sterile water and the diluted collection went through a C-18 Sep-Pak Plus cartridge to trap the 2-[^18^F]fluoroethyl tosylate on the Sep-Pak. The trapped product was eluted with diethyl ether (2.5 mL).

##### 2.2.2.3. Radiosynthesis of [^18^F]**2b**

The eluted solution formed two phases: the top ether phase was transferred out, and the bottom aqueous phase was extracted with another 1-mL aliquot of ether. The combined ether extracts were passed through a set of two sodium sulfate Sep-Pak Plus dry cartridges into a reaction vessel. After ether was evaporated with a nitrogen stream at 25 °C, 1.0 mg of Precursor **4** was dissolved in 200 μL DMSO and was transferred to a vial containing 1–2 mg Cs_2_CO_3_. After vortexing for 1 min, the Cs_2_CO_3_ saturated solution was added to the reaction vessel containing the dried radioactive [^18^F]/F^−^. The tube was capped and briefly vortexed and then kept at 90 °C for 15 min. Ten microliters of 1,8-diazabicyclo[5.4.0]undec-7-ene (DBU) in 50 μL DMSO was added via syringe. The reaction mixture was heated at 90 °C for 15 min. The mixture was subsequently diluted with 3 mL of the HPLC mobile phase (50/50 acetonitrile/0.1 M formate buffer, pH = 4.5) and purified using a Semi-Prep HPLC system for purification. The HPLC system contains a 5-mL injection loop, an Agilent SB C-18 column, a UV detector at 254 nm and a radioactivity detector. At a 4.0 mL/min flow rate, the retention time of the product was 19–21 min, whereas the retention time of the precursor was 8–9 min. After the HPLC collection and being diluted with 50 mL sterile water, the product was trapped on a C18 Sep-Pak Plus cartridge. The product was eluted with ethanol (0.6 mL) followed by 5.4 mL 0.9% saline. After sterile filtration, the final product was ready for quality control (QC) analysis and animal studies. An aliquot was assayed by analytical HPLC (Grace Altima C18 column, 250 × 4.6 mm), UV at 276 nm, mobile phase of acetonitrile/0.1 M ammonium formate buffer (71/29, *v*/*v*), pH = 4.5. Under these conditions, the retention time for [^18^F]**2b** was approximately 4.9 min at a flow rate of 1.5 mL/min. The sample was authenticated by co-injecting with the cold standard **2b** solution. The radiochemical purity was >98%; the chemical purity was >95%; the labeling yield was 55%–65% (*n* = 4, decay corrected), and the specific activity was >200 GBq/μmol (decay corrected to EOB, *n* = 4).

### 2.3. Biodistribution Studies

All animal experiments were conducted in compliance with the Guidelines for the Care and Use of Research Animals under protocols approved by Washington University's Animal Studies Committee. For the biodistribution studies, 11.1–13.0 MBq of [^11^C]**2a** in 200–250 μL or 1.67–1.85 MBq of [^18^F]**2b** in 200–250 μL of saline containing 10% ethanol was injected via the tail vein into mature male Sprague-Dawley rats (175–240 g) under 2%–3% isoflurane/oxygen anesthesia. Group of rats (*n* = 4) were used for each time point. At 5, 30, 60 min (and 120 min for [^18^F]**2b**) post injection, the rats were anesthetized and euthanized. The whole brain was quickly removed and dissected into segments consisting of brain stem, thalamus, striatum, hippocampus, cortex and cerebellum. The remainder of the brain was also collected to determine total brain uptake. At the same time, samples of blood, heart, lung, muscle, fat, pancreas, spleen, kidney, liver (and bone for [^18^F]**2b**) were removed and counted in a Beckman Gamma 8000 well counter with a standard dilution of the injectate. Tissues were weighed, and the percent injected dose (%ID)/gram for each tissue was calculated.

### 2.4. MicroPET Brain Imaging Studies of [^11^C]**2a** in Cynomolgus Macaque

Following the initial evaluation in rats, the washout kinetics and ability of [^11^C]**2a** to cross the blood-brain barrier in a non-human primate (NHP) was determined on an adult male cynomolgus macaque (6–8 kg) using a microPET Focus 220 scanner (Concorde/CTI/Siemens Microsystems, Knoxville, TN). Before each scan (*n* = 2), the animal was fasted for 12 h and then initially anesthetized with ketamine (10 mg/kg) and glycopyrrolate (0.13 mg/kg) intramuscularly. Upon arrival at the scanner, the monkey was intubated, and a percutaneous catheter placed for tracer injection. The head was positioned supine in the adjustable head holder with the brain in the center of the field of view. Anesthesia was maintained at 0.75%–2.0% isoflurane/oxygen and the core temperature maintained at 37 °C. A 10-min transmission scan was performed to confirm positioning; this was followed by a 45-min transmission scan for attenuation correction. Subsequently, a 2-h dynamic emission scan was acquired after venous injection of 300-370 MBq of [^11^C]**2a**.

## 3. Results and Discussions

### 3.1. Chemistry

Compounds **2a** and **2b** possess a methoxy and fluoroethoxy group, respectively, enabling radiolabeling through *O*-alkylation of the corresponding phenol precursor. However, to avoid undesired *N*-alkylation product, the acetyl protected Compound **4** was used as the precursor for the radiosyntheses. As shown in Scheme 1, the synthesis of **4** was accomplished by a two-step strategy starting from **2a** following our previous procedure [17]. *N*-acetylation of **2a** was achieved using acetyl chloride. Removing the *O*-methyl group of **3** with boron tribromide afforded the phenol Precursor **4**, which was used in the radiosyntheses of **2a** and **2b**. Due to the reaction scale difference, the yields for certain reactions differ slightly from our previous report.

### 3.2. Radiochemistry

The radiosynthesis of [^11^C]**2a** was accomplished by a two-step approach. The reaction of the phenol Precursor **4** with [^11^C]CH_3_I was performed in DMF in the presence of NaOH [19–21], and the *N*-acetyl group of the ^11^C-labeled intermediate was removed by DBU following the literature procedure [22], as outlined in Scheme 2. [^11^C]**2a** was obtained in approximately 35%–45% overall radiochemical yield (RCY) after HPLC purification (*n* = 4). The radiochemical purity of [^11^C]**2a** was >99% and chemical purity was >95%. [^11^C]**2a** was identified by co-eluting with the solution of standard **2a**. The entire synthetic procedure, including the production of [^11^C]CH_3_I, radiosynthesis, HPLC purification and formulation of the radiotracer for animal studies, was completed within 50–60 min. [^11^C]**2a** was obtained in a specific activity of >363 GBq/μmol at EOB (*n* = 4).

The radiosynthesis of [^18^F]**2b** was achieved using a three-step reaction. The radioactive intermediate, [^18^F]fluoroethyltosylate ([^18^F]FEOTs), was first synthesized through a typical fluorination of the di-tosylate substrate [23–27]. Treatment of ethylene glycol ditosylate using [^18^F]fluoride, potassium carbonate and Kryptofix 222 gave [^18^F]FEOTs in good yield (60%–70%, decay corrected) after HPLC purification. The intermediate was reacted with the precursor, followed by DBU hydrolysis to afford a sufficient dose of [^18^F]**2b** with the labeling yield of 55%–65% after HPLC purification (*n* = 4, decay corrected to EOB). The radiochemical purity of [^18^F]**2b** was >98%, and the chemical purity was >95%. [^18^F]**2b** was identified by co-eluting with the solution of standard **2b**. The entire synthetic procedure, including the drying of [^18^F]F^−^, the radiosynthesis, HPLC purification and formulation of the radiotracer for *in vivo* studies, was completed in 3 h. Radiotracer [^18^F]**2b** was obtained in a specific activity of >200 GBq/μmol (decay corrected to EOB, *n* = 4).

### 3.3. Biodistribution in Rats

The radioactivity distribution in various organs after the injection of [^11^C]**2a** and [^18^F]**2b** in rats is summarized in Table 1. Both radiotracers displayed homogeneous distribution in the brain regions as shown in Figure 2A,B. For [^11^C]**2a**, the total brain uptake (%ID/gram) of radioactivity at five, 30 and 60 min post injection were 0.953 ± 0.115, 0.287 ± 0.046 and 0.158 ± 0.014 respectively; in the peripheral tissues, liver had the highest uptake among the tissues analyzed; the uptake (%ID/gram) in liver reached 2.198 ± 0.111 at 5 min and 1.116 ± 0.024 at 60 min. For [^18^F]**2b**, the total brain uptake (%ID/gram) at five, 30, 60 and 120 min was 0.758 ± 0.013, 0.465 ± 0.018, 0.410 ± 0.030 and 0.359 ± 0.016, respectively; At 5 min post-injection, this compound also has a high liver uptake: (%ID/gram) of 1.626 ± 0.221. However, after 30 min, the kidney has retained the highest radioactivity of all tissues that were analyzed. The bone uptake (%ID/gram) was very stable and no defluorination was observed for [^18^F]**2b**. More importantly, the *ex vivo* rat biodistribution data revealed that both compounds readily crossed the BBB and entered the brain. Both tracers exhibit high initial brain uptake and appropriate washout kinetics in the brain of normal rats. Rapid clearance of the radioactivity for both [^11^C]**2a** and [^18^F]**2b** was observed from brain, as well as other organs, such as lung, pancreas, spleen, kidney and liver. However, [^11^C]**2a** showed faster wash-out kinetics than [^18^F]**2b**, as shown in Figure 2. [^11^C]**2a** was chosen for subsequent microPET evaluation in an NHP.

### 3.4. MicroPET Studies in NHP

**Figure 3:**
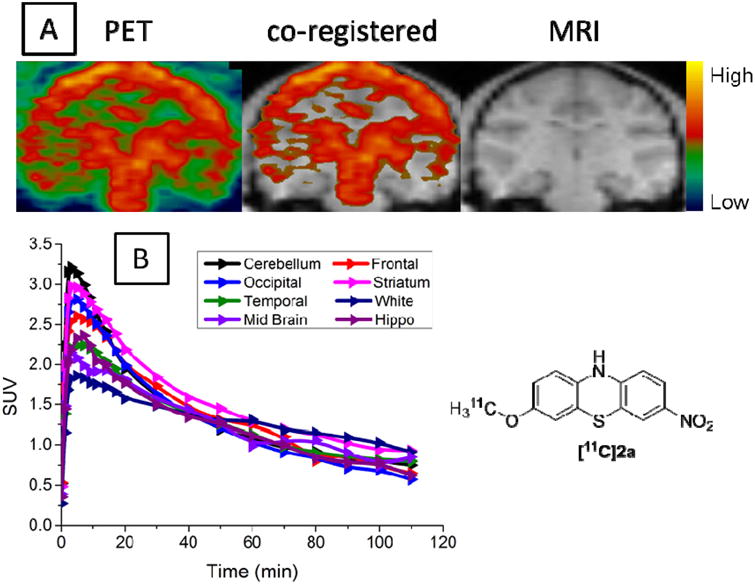
*In vivo* microPET brain imaging studies of [^11^C]**2a** in cynomolgus macaque. (**A**) PET image (**left**), co-registered with MRI (**middle**), MRI image (**right**); (**B**) time-activity curves. SUV, standardized uptake value.

The representative summed images from zero to 120 min were co-registered with MRI images to accurately identify the regions of interest (Figure 3). The time-activity curve (TAC) revealed high initial uptake of [^11^C]**2a** in the brain, which peaked at 3 min post-injection; then, the radioactivity was quickly washed out from all brain regions. The summed image revealed a homogeneous distribution of radioactivity in the brain of the normal cynomolgus macaque. The microPET studies suggested that [^11^C]**2a** was able to cross the BBB and had a fast washout kinetics from the brain regions. The macaque used in the studies was a healthy young adult, and the distribution of the α-syn radioligand throughout the brain regions was homogeneous. Higher expression of α-syn protein in particular regions should not be observed in healthy subjects; thus, homogeneous distribution of the radioactivity in the macaque brain was expected. Nevertheless, PET studies of [^11^C]**2a** performed on an NHP model bearing the over-expression of α-syn aggregation will directly determine the *in vivo* specificity of the radiotracer.

## 4. Conclusions

In summary, two α-syn ligands, [^11^C]**2a** and [^18^F]**2b**, were successfully radiosynthesized by *O*-alkylation of the desalkyl precursor. The biodistribution studies of [^11^C]**2a** and [^18^F]**2b** were conducted in male Sprague-Dawley rats and showed that both tracers were able to cross the BBB with high initial uptake. At 5 min post-injection, the uptake (%ID/gram) reached 0.953 ± 0.115 for [^11^C]**2a** and 0.758 ± 0.013 for [^18^F]**2b**. Both [^11^C]**2a** and [^18^F]**2b** displayed a homogeneous distribution throughout the brain of healthy adult male rats with rapid washout kinetics. *In vivo* microPET imaging in a healthy cynomolgus macaque confirmed that [^11^C]**2a** was able to enter the brain, had homogeneous distribution and rapid washout kinetics. Further structural optimization of [^11^C]**2a** and [^18^F]**2b** may lead to a highly specific radioligand for imaging α-syn aggregation *in vivo*.

## Figures and Tables

**Table 1:** Biodistribution of [^11^C]**2a** and [^18^F]**2b** in male Sprague-Dawley rats (%ID/gram).

Radioligand	Organ	5 min	30 min	60 min	120 min
[^11^C]**2a**	blood	0.506 ± 0.040	0.369 ± 0.031	0.300 ± 0.015	
	heart	0.758 ± 0.052	0.377 ± 0.046	0.245 ± 0.010	
	lung	1.149 ± 0.058	0.740 ± 0.038	0.485 ± 0.036	
	muscle	0.271 ± 0.005	0.325 ± 0.030	0.199 ± 0.016	
	fat	0.155 ± 0.023	0.241 ± 0.042	0.293 ± 0.076	
	pancreas	1.007 ± 0.262	0.506 ± 0.066	0.522 ± 0.036	
	spleen	0.659 ± 0.049	0.400 ± 0.052	0.386 ± 0.027	
	kidney	1.362 ± 0.054	0.807 ± 0.086	0.559 ± 0.053	
	liver	2.198 ± 0.111	1.349 ± 0.116	1.116 ± 0.024	
	brain	0.953 ± 0.115	0.287 ± 0.046	0.158 ± 0.013	

[^18^F]**2b**	blood	0.553 ± 0.047	0.589 ± 0.016	0.606 ± 0.035	0.585 ± 0.046
	heart	0.757 ± 0.033	0.505 ± 0.015	0.466 ± 0.040	0.410 ± 0.030
	lung	0.833 ± 0.053	0.561 ± 0.021	0.491 ± 0.030	0.436 ± 0.018
	muscle	0.430 ± 0.031	0.451 ± 0.018	0.376 ± 0.019	0.315 ± 0.015
	fat	0.255 ± 0.037	0.425 ± 0.023	0.371 ± 0.067	0.293 ± 0.048
	pancreas	1.004 ± 0.147	0.546 ± 0.068	0.409 ± 0.029	0.330 ± 0.021
	spleen	0.672 ± 0.064	0.509 ± 0.022	0.446 ± 0.038	0.398 ± 0.019
	kidney	1.070 ± 0.058	0.988 ± 0.090	0.678 ± 0.032	0.659 ± 0.027
	liver	1.626 ± 0.221	0.847 ± 0.027	0.561 ± 0.028	0.467 ± 0.023
	bone	0.340 ± 0.027	0.309 ± 0.020	0.407 ± 0.043	0.644 ± 0.071
	brain	0.758 ± 0.013	0.465 ± 0.018	0.410 ± 0.030	0.359 ± 0.016

## Abbreviations

α-syn: α-synuclein
BBB: blood-brain barrier
calcd: calculated
CNS: central nervous system
DBU: 1,8-diazabicyclo[5.4.0]undec-7-ene
DCM: dichloromethane
DMF: *N*,*N*-dimethylformamide
DMSO: dimethyl sulfoxide
EOB: end of bombardment
[^18^F]FEOTs: [^18^F]fluoroethyltosylate
HPLC: high performance liquid chromatography
HRMS: high resolution mass spectrometry
LBs: Lewy bodies
LNs: Lewy neuritis
NHP: non-human primate
PD: Parkinson's disease
PET: positron emission tomography
QC: quality control
RCY: radiochemical yield
SAR: structure-activity relationship
SD: Sprague Dawley
SUV: standardized uptake value
TAC: time-activity curve
VMAT2: vesicular monoamine transporter type 2
